# The Open SprayBot: A high-throughput paper spray mass spectrometry platform for disease screening

**DOI:** 10.1016/j.ohx.2024.e00551

**Published:** 2024-06-30

**Authors:** Nancy Shyrley García-Rojas, Héctor Guillén-Alonso, Scott MacKay, Claudia Torres-Calzada, Leonardo Daniel Soto-Rodriguez, Robert Winkler, David S. Wishart

**Affiliations:** a Laboratorio de Análisis Bioquímico e Instrumental, Unidad de Genómica Avanzada, Cinvestav, Km. 9.6 Libramiento Norte, Carretera Irapuato-León, Irapuato, Guanajuato, México; b Department of Biochemical Engineering, National Technological Institute, Celaya, Mexico; c Department of Biological Science, University of Alberta, Edmonton, Canada; d Department of Computing Science, University of Alberta, Edmonton, Canada

**Keywords:** Paper-spray mass spectrometry, Open-source, 3D-printing, High-throughput

## Abstract

Newborn disease screening increases survival, improves quality of life and reduces treatment costs for healthcare systems. Mass spectrometry (MS) is an effective method for metabolic screening. However, conventional analytical methods require biofluid handling and cooling conditions during transport, making the logistics difficult and expensive, especially for remote regions. ’Paper-spray’ (PS) ionization generates a charged solvent spray from samples deposited on paper strips. Therefore, samples can be applied on a suitable matrix and shipped as dried spots to diagnostic laboratories with standard postal or messenger services. We built a robotic platform, the ’Open SprayBot’, to automatically analyze paper-deposited samples via PS-MS and increase the sample throughput. The system is operated via RUMBA32 and Arduino Mega boards. A commercial syringe pump and power supply provide solvent application and electrical current required for PS-MS. The usability of the Open SprayBot was demonstrated by quantifying palmitoyl-l-carnitine, a common biomarker in newborn screening.

## Graphical abstract

**Figure:**
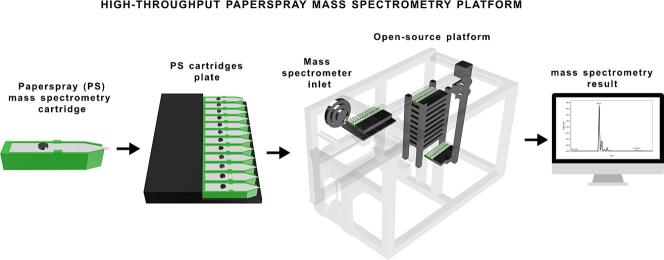

Specification table

**Table:** 

Hardware name	The Open SprayBot
Subject area	Open-source alternatives to existing infrastructure
Closest commercial analog	Verispray (Thermo-Fisher Scientific, USA)
Hardware type	Ambient ionization mass spectrometry sampling platform
Open-source license	Attribution-NonCommercial-ShareAlike 4.0 International(CC-BY-NC-SA 4.0)
Cost of hardware	$ 6801 CAD
Source file repository	https://zenodo.org/doi/10.5281/zenodo.8083881

## 1 Hardware in context

Early disease detection is essential for cost-effective public health systems. The primary methods for disease screening involve biofluid testing and medical imaging. However, the selection of a screening test is influenced by factors including accessibility, cost-effectiveness, and the specific disease being screened. Opting for a more expensive test can often be the ideal approach if it proves to be effective. A case in point is newborn screening for inborn errors of metabolism (IEM). Newborn screening is a very effective public health program established in many countries worldwide. IEMs are typically screened by lancing a baby’s foot, collecting a blood spot on a paper card, and submitting it for mass spectrometry (MS) analysis. MS is preferred due to its sensitivity, relatively rapid turn-around and low false-negative rate [1], [2]. The development of improved analytical methods and better MS instrumentation has helped make IEM disease screening far more sensitive [3], [4], [5]. However, the sample work-up required for blood spots is still tedious and time-consuming and much work still needs to be done in this area to reduce time and costs [6], [7], [8].

Ambient ionization mass spectrometry (AIMS) techniques have opened a window to potentially expedite MS-based newborn screening. AIMS requires minimal sample preparation, allowing biofluids such as blood or urine to be analyzed in minutes [1], [2], [3]. In this regard, paper-spray mass spectrometry (PS-MS) is an ambient ionization method that has proven to be particularly fast and ideal for working with blood or urine deposited on filter paper [5], [8], [9], [10]. These characteristics make PS-MS an ideal candidate for eliminating the costly, time-consuming sample preparation steps in newborn screening or other relevant clinical areas such as drug screening.

PS-MS requires that the sample of interest be deposited on a piece of paper (approximately 5 x 10 mm) and cut in such a way as to have a small triangular tip. This tip must be aligned with the mass spectrometer’s inlet, which is typically only a few micrometers in diameter. Therefore, precise alignment is essential. Additionally, the ionization process requires the injection of a solvent onto the paper followed by the application of a high electric potential to induce ionization.

To streamline the PS-MS process, several useful approaches have been developed, mostly within the research community [11], [12]. These efforts have spurred the commercialization of high-throughput (HT) instrumentation for PS-MS [10], [13]. One example is the Verispray platform from Thermo Fisher Scientific (CA, USA). This instrument comprises two modules. The first module is the ion source, which includes space for a sample plate – available from the same vendor − that can accommodate up to 24 samples. The second optional module consists of a plate loader and magazine with the capacity to hold up to 10 sample plates, allowing for the storage of up to 240 samples. While this development enables HT PS-MS, it is very expensive, fully proprietary, and vendor-specific.

To enable HT PS-MS on any MS platform our group has developed a low-cost HT PS-MS system that can, in principle, work on any MS instrument and which can be easily adapted to enable PS-MS research or testing in appropriately equipped MS labs. This platform could also be used to accelerate drug testing or newborn disease screening via PS-MS in accredited MS testing labs.

This work is grounded in using open-source technologies, low-cost (generic) equipment, and parts made via 3D printers which are widely accessible in many research labs. Indeed, 3D-printing technologies have catalyzed the rapid development of many tools across various research fields and industries, including AIMS [14], [15], [16].

## 2 Hardware description

This HT PS-MS platform is built upon open-source technologies, utilizing 3D printing, and incorporates open-source hardware, control boards, and software as well as low-cost (generic) equipment or materials. A previous version developed by our group, known as “The Open LabBot”, enabled MS analysis using several ambient ionization techniques [14], [17], [18] but not by PS-MS. This new platform has been redesigned to automate the analysis of blood or urine using PS-MS and is now referred to as “The Open SprayBot”. The primary goal of the platform is to enable the HT PS-MS analysis of blood or urine samples deposited on filter papers. These filter papers can be easily cut into paper strips with a triangular tip (via a paper-cutting dye), and then inserted into 3D-printed cartridges and placed in a 3D-printed tray to facilitate their automated manipulation. Each tray can hold a maximum of 12 cartridges and, each cartridge contains a piece of filter paper with a triangle tip. While commercial PS-MS plates are typically designed to hold 24 cartridges. This design is more compatible with open-access 3D printer technology, making it an affordable option for use with any MS platform. To increase capacity, a shelf for 10 trays was included, allowing for the processing of up to 120 samples in a single analytical run. Despite having less capacity than commercial instruments (240 samples), this system is significantly cheaper, costing approximately 20 times less [19].

Other lab-built platforms for PS-MS described in the literature still require manual intervention for sample exchange [11], [20]. In contrast, this platform eliminates the need for manual sample exchange, accelerating the PS-MS analysis and making it a true HT platform.

Overall, this open-source platform for HT PS-MS offers:

• An affordable, all-in-one alternative to commercial systems for HT analysis by PS-MS that is vendor-independent.
• The capability to 3D-print many of the key components and consumables, making replacements inexpensive and repairs easy to do.
• Easily replaceable parts for enhanced convenience.

The system was designed and built in collaboration with MakerMex Co. (Mexico). The firmware employed for the RUMBA board is Marlin (version 3), an open-source software developed in 2011 for 3D-printers (https://marlinfw.org) and modified by MakerMex. The software is included in the repository for both the RUMBA32 and the Arduino Mega 2560. Testing experiments of the system were made with chromatographic paper Whatman #1 (Sigma-Aldrich, USA).

This HT PS-MS platform includes both a sampling module and an ionization module and is compatible with many MS instruments. These modules are described in more detail below.

### 2.1 Sampling module

**Fig. 1:**
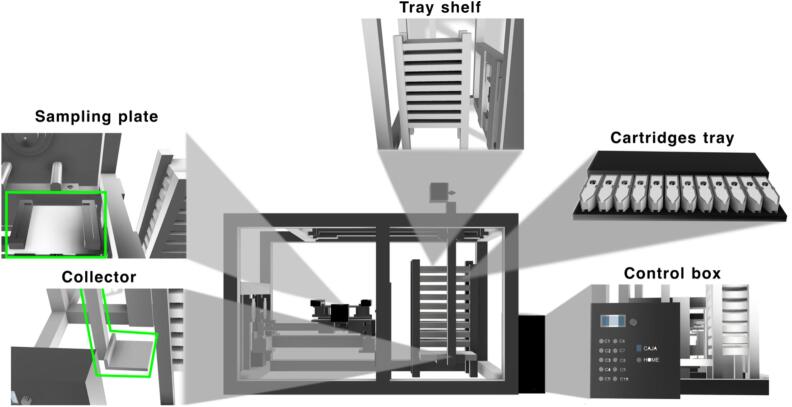
The Open SprayBot platform. Representation of the modules for PS-MS analysis.

The sampling module (Fig. 1) comprises a sampling plate (with a capacity for 12 PS cartridges) that allows to align of each PS cartridge with the mass spectrometer inlet and facilitates automated sample exchange. Additionally, the sampling module includes a tray shelf with the capacity to accommodate up to 10 separate trays. The sampling module includes a collector that retrieves the trays from the shelf and places them onto the sampling plate. The collector can move along the X, Y, and Z axes.

**Fig. 2:**
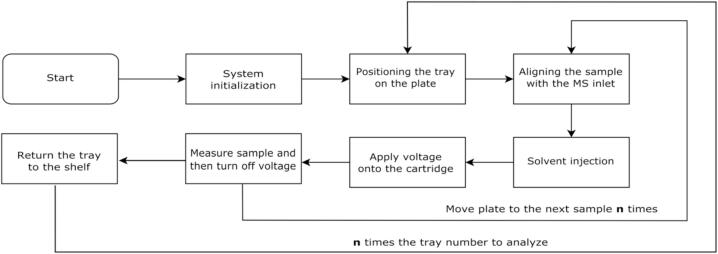
Summary of the operating workflow. n: number of samples or trays customizable.

The operational workflow of the system is presented in Fig. 2. The number of samples and the number of trays can be modified according to the user's specifications.

### 2.2 Ionization module

Following the alignment of the cartridge with the paper strip with the mass spectrometer inlet, the next step involves ionization. The solvent is first injected onto the paper via the PEEK (a high-strength thermoplastic) tubing connected to a syringe. Once the solvent is applied to the paper a high-voltage is applied to the paper via a high-voltage source, the sample molecules are then ionized and detected by the mass spectrometer. After the sampling, the voltage must be turned off to prepare the platform for the analysis of the next sample. A flowchart depicting the operational process of the ionization and analysis process is shown in Fig. 2.

## 3 Design files summary

### 3.1 Printing recommendations

Acetal/Delrin filament is recommended as the 3D printing material for the cartridges as the chemical noise is lower. PLA is suitable for the remaining 3D-printed parts. The infill density in the cartridge tray must be at least 30 % to provide sufficient weight for the collector to lift and place the tray efficiently.

## 4 Bill of materials summary

Table 2.

## 5 Build instructions

The complete system is composed of four main sections: 1) The sampling platform, 2) PS-MS cartridges, 3) the ionization module, and 4) the solvent handling mechanism. The assembling instructions were divided into these four sections.

### 5.1 Assembly of the main structure: Collector and sampling plate

**Fig. 3:**
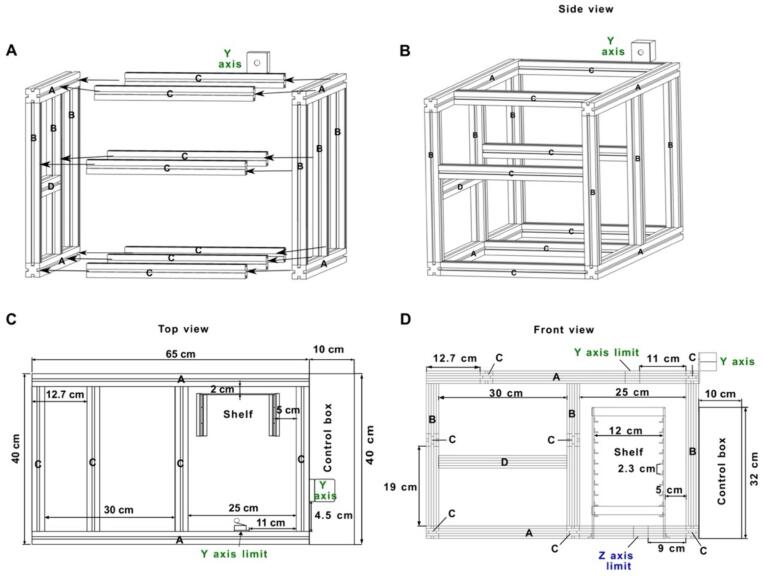
Assembling the structure. A) assembling the main structure B) side view C) top view D) front view.

**Fig. 4:**
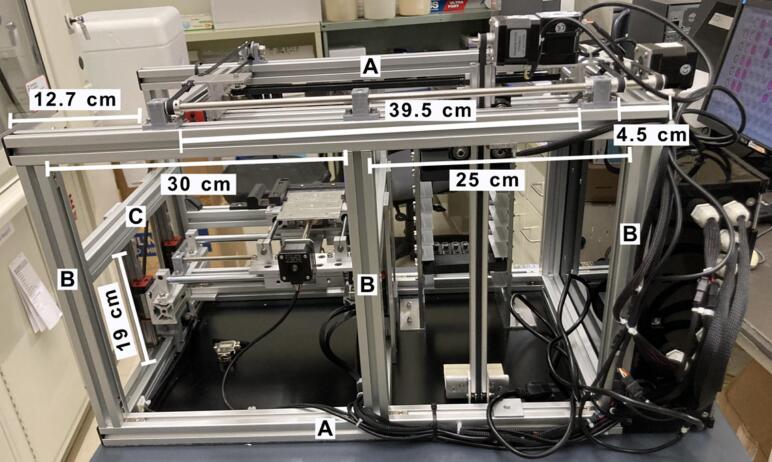
The Open SprayBot platform. Distribution dimensions are shown in the front view of the system.

The platform includes two key components: the collector and the sampling plate. Fig. 3 provides a detailed guide for assembling the main structure using the extruded frame components listed in Table 2. The accurate positioning of the shelf is essential for the precise lifting process of the PS-MS trays. The position of the shelf in Fig. 3C and Fig. 3D is depicted in relation with the frame parts **A** and **C**, and **A** and **B**, respectively. The shelf and the frame structure are screwed to the supporting plate, which is included in Table 2 as the aluminum composite sheet. The position distance for assembling the main structure is shown in Fig. 3 and Fig. 4.

**Fig. 5:**
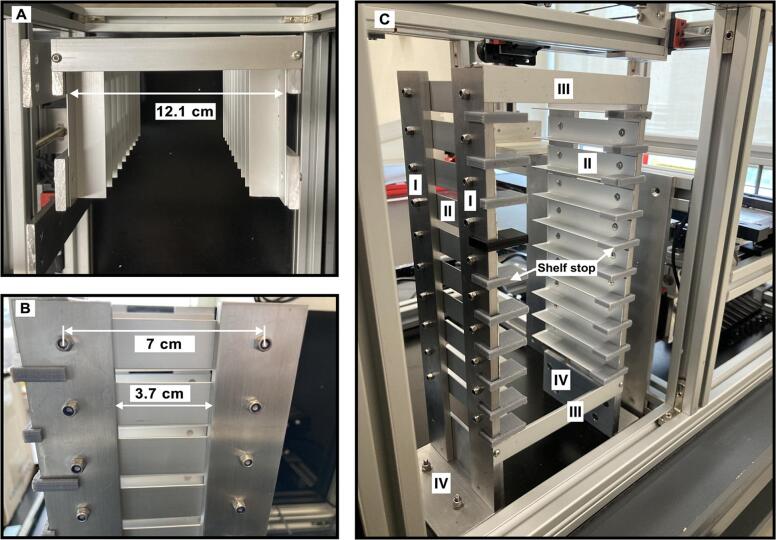
Trays’ shelf of the open SprayBot platform. A) top view, B) lateral view and C) isometrical view.

Fig. 5 shows the assembled shelf along with its components, which are included in Table 2. For instance, Fig. 5A depicts the required distance between the aluminum angles II.

Such space on the shelf is necessary for storing the cartridges tray on the shelf.

**Fig. 6:**
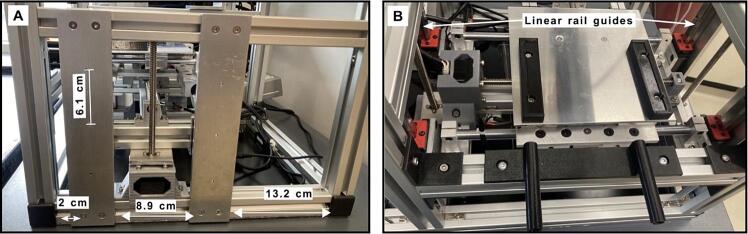
Sampling plate. A) Position of the lateral flat bars supporting the sampling plate, B) The linear rail guides of the sampling plate are secured by these supporting bars.

Fig. 6A and Fig. 6B illustrate the placement of the flat bars that support the sampling plate through two mini linear guides, with each side attached by two screws. See Fig. 6.

In Fig. 5C the position of the shelf top (3D printed) is illustrated, as well as the distance of the screws to join the aluminum angles II to the aluminum bars I.

In **A** the position of the aluminum flat bars XIV is showed. Such position is regarding frame C, at 2 cm of distance from frame B, next to frame D.

A. **Collector assembly.**

Fig. 3C and Fig. 3D illustrate the placement of the Y-axis motor (highlighted in green) of the collector within the main structure. The motors for the X and Z axes of the collector, which are mobile, are shown in Fig. 8A.

**Fig. 7:**
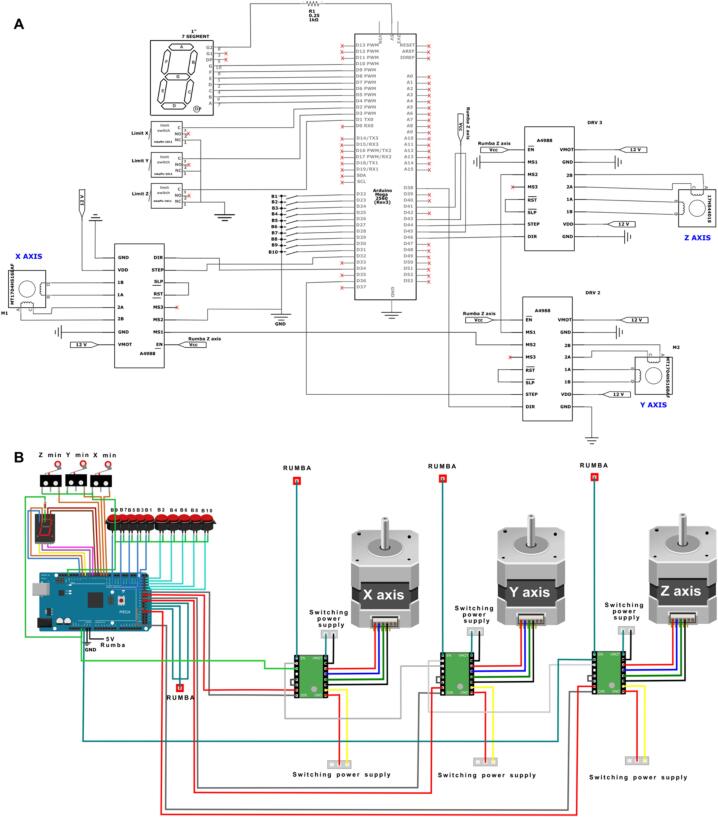
Collector electronics. A) The circuit scheme for the collector controlled by the Arduino Mega 2560 board and B) configuration of the components controlling the collector section, including 3 stepper drivers A4988 controlling the stepper motors.

**Fig. 8:**
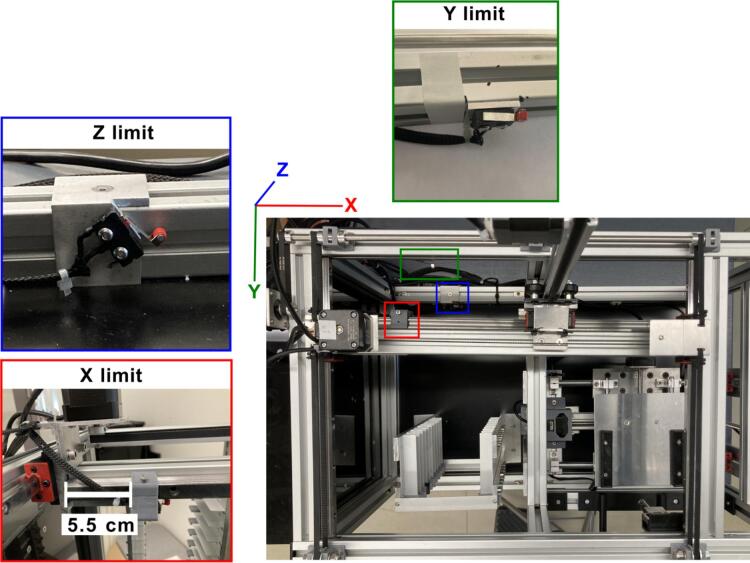
Position of the axes limit switches for the collector. Switch for the x-axis limit in red, Switch for the y-axis limit in green and, switch for the z-axis limit in blue. (For interpretation of the references to colour in this figure legend, the reader is referred to the web version of this article.)

**Fig. 9:**
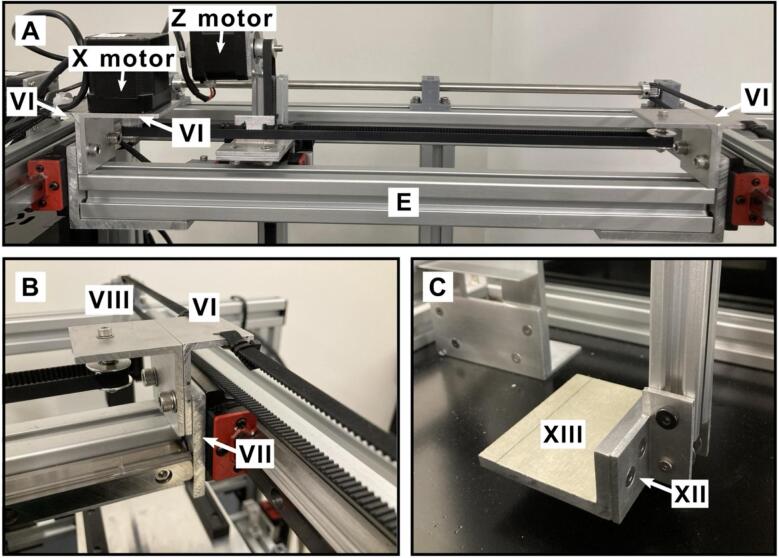
Assembly of the collector and collector support. A) and B) show the X-axis support configuration and, C) illustrates the tray collector.

The Z-axis motor is attached to the T-slot frame 2020 (collector frame), while the X-axis motor is attached to the E frame (Fig. 9). The Y-axis limit switch is shown in both, Fig. 3C and Fig. 3D. The position of the Z-axis limit switch is shown in Fig. 3D (highlighted in blue) and in Both Y and Z limit switches are held by an aluminum angle, as included in Table 2 and depicted in Fig. 8. The X-axis limit switch is held by a 3D-printed supports listed in Table 1 as *limit switch holder 1* and *2*. These 3D printed parts are attached to the mini linear rail guide of the aluminum frame **E** (Fig. 8) at 5.5 cm from the linear rail guide as shown in Fig. 8 highlighted in red. The position of the limit switches for the Y and Z axes of the collector is given in Fig. 3 and Fig. 8.

**Table 1:** Design files summary. ^a^ The quantity is for maximum capacity, and it depends on the user’s needs. Free for non-commercial use (CC-BY-NC-SA 4.0). ^b^ designs available in zenodo repository: https://zenodo.org/doi/10.5281/zenodo.8083881.

**Part**	**Pieces**	**File name**	**Picture**	**File type^b^**	**Description**
A1	120^a^	PS-MS-cartridge		STL, F3D	Contains the filter paper for paper-spray analysis
A2	10^b^	Cartridge-tray		STL, F3D	Contains the cartridges during the analysis
B1	2	Tray-support		STL, F3D	Holds the tray during the analysis
B2	2	Z-axis-motor-support		STL, F3D	Holds the 2 stepper motors controlling the z-axis of the sampling plate
B3	1	X-axis-motor-support		STL, F3D	Support for the stepper motors controlling the x-axis of the sampling plate.
B4	2	Threaded-shaft-sampling-plate		STL, F3D	Holds the threaded shaft of the x-axis on the sampling plate.
B5	1	MS-source-guides		STL, F3D	Assists in positioning the plate in front of the mass spectrometer at a constant distance.
B6	4	Union-extruded-frame-d		STL, F3D	Joins the D frame with the B frame
B7	1	Leadscrew-nut-support-X-axis		STL, F3D	Joins the sampling plate to the leadscrew of the x-axis
B8	1	Leadscrew-nut-support-Y-axis		STL, F3D	Joins the sampling plate to the leadscrew of the y-axis
C1	3	Shaft-support-collector		STL, F3D	Support of the shaft of the linear rods of the collector axes
C2	2	Limit-switch-holder1		STL, F3D	Holds the limit switch for 2 axes, the x-axis of the collector and the z-axis of the sampling plate (part 1)
C3	2	Limit-switch–holder2		STL, F3D	Holds the limit switch for 2 axes, the x-axis of the collector and the z-axis of the sampling plate (part 2)
C4	2	Belt-307A-support-collector		STL, F3D	Holds the pulley wheels for the y-axis of the collector
D1	2	Control-box-screw-support		STL, F3D	Aids in closing the door of the control box
D2	1	Doorhandle		STL, F3D	Component of the control box
E1	1	MS-Interlock-bar		STL, F3D	Pushes the interlock of the mass spectrometer
E2	10	Shelf-stop1		STL, F3D	Prevents the tray from falling or moving from the shelf part 1)
E3	10	Shelf-stop2		STL, F3D	Prevents the tray from falling or moving from the shelf (part 2)
S1	1	Spring-mechanism- core-part1		STL, F3D	Contains the 2 springs
S2	1	Spring-mechanism- core-part2		STL, F3D	Assists in compressing and extending the springs
S3	1	Cable-guide-part1		STL, F3D	Guides the electrical cable
S4	1	Cable-guide-part2		STL, F3D	Guides both the electrical cable and solvent tubing
S5	1	Spring-mechanism-attachment-support		STL, F3D	Attaches and joints the spring mechanism to the extruded aluminum frame A
S6	1	Spring-mechanism-holder		STL, F3D	Contains the core of the spring mechanism
S7	1	Springs-lock		STL, F3D	Secures the core of the spring mechanism in the spring mechanism holder

The 3 stepper motors of the collector are controlled using an Arduino Mega 2650 board. The stepper motors are powered at 24 V. A wiring diagram and the schematic circuit for the collector section are shown in (Fig. 7*)*. The code to control the system is included in the zenodo repository (https://zenodo.org/doi/10.5281/zenodo.8083881).

B. **Assembling the sampling plate**.

**Fig. 10:**
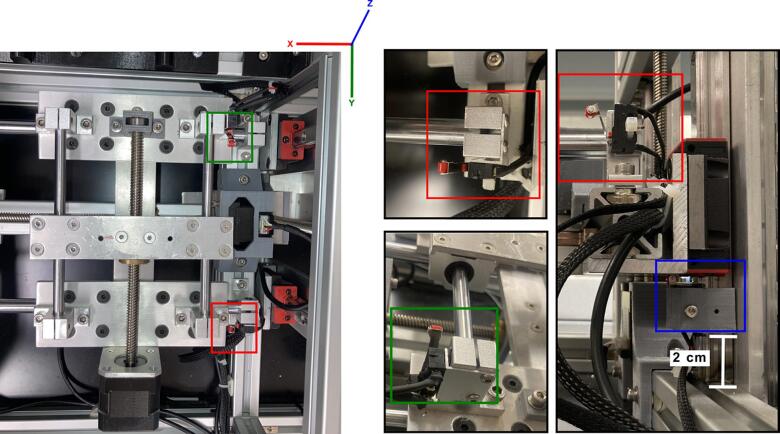
Position of the limit switches of the sampling plate. Switch for the x-axis limit in red, Switch for the y-axis limit in green and Switch for the z-axis limit in blue. (For interpretation of the references to colour in this figure legend, the reader is referred to the web version of this article.)

**Fig. 11:**
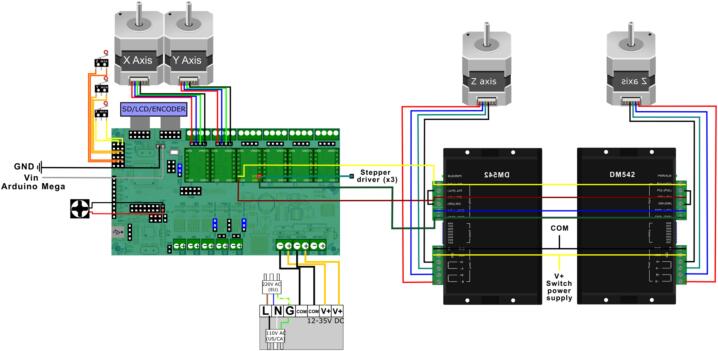
Configuration of the Rumba32 board and the parts controlling the sampling plate.

The sampling plate is supported by the flat bars shown in. The two stepper motors for the Z-axis are placed between these flat bars on each side and are attached to the frame C using the 3D-printed support listed in Table 1. The positions of the limit switches for the sampling plate are depicted in Fig. 10. The sampling plate was operated by a Rumba32 board. This board was designed for 3D printers for components such as the extruder and the printing bed. Fig. 11 illustrates the configurations of the Rumba32′s pin output for controlling the sampling plate.

Two stepper motor drivers (DM542) were incorporated to ensure smooth and precise movement of the sampling plate along the Z-axis.

### 5.2 Cartridges for PS-MS

**Fig. 12:**
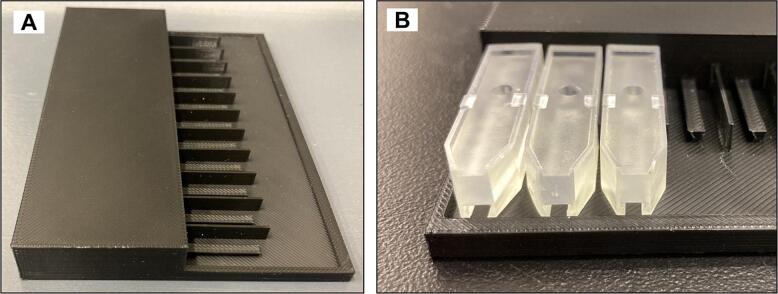
PS-MS cartridges. A) cartridges tray and B) resin cartridges.

PLA filaments were used in a Prusa i3 Mk3/Mk3s printer to make the cartridges tray, and standard resin was used in a Formlab printer for printing the cartridges (Fig. 12).

### 5.3 Ionization process

**Fig. 13:**
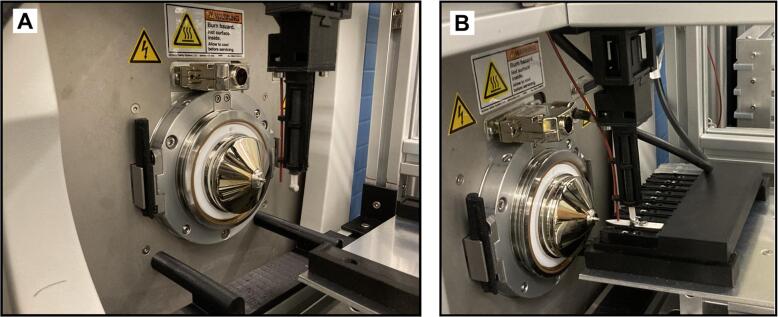
View of the system in front of a mass spectrometer inlet. A) the 3D printed guides align with the pin guides of the mass spectrometer, and B) a cartridge is aligned with the sampling plate.

**Fig. 14:**
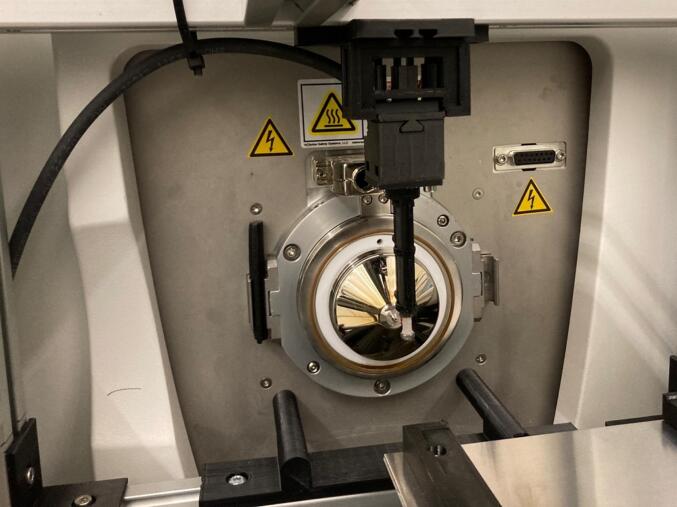
Setup of the mass spectrometer inlet.

An external power supply was incorporated to regulate the voltage, and a syringe pump was used to inject the ionization solvent. The pump is controlled using the software of the mass spectrometer. Additionally, a PEEK tubing support system was included to facilitate the solvent injection (Fig. 13). The voltage is applied via a coaxial cable included with the external power supply. A spring mechanism system 3D printed in PLA holds the coaxial cable. The spring mechanism is shown in Fig. 13 and Fig. 14, and is attached to an extruded aluminum A as shown in Fig. 14**.**

## 6 Operation instructions

Place the system in front of the mass spectrometer inlet ensuring that the 3D printed guides are aligned with the ion source housing guide pins of the mass spectrometer, as shown in Fig. 14.

The platform operates on a 120 V connection, with the control done using the control knob in the control box. Additionally, the platform can be controlled via G-code commands using the USB port of the Rumba board in Arduino software. To start operating the platform, the initial position for both the collector and the sampling plate was first set. This is done by selecting the auto-home function for the sampling plate using the control knob and pressing the “home” button for the collector. The trays containing the cartridges are inserted onto the shelf for analysis. Each tray can be removed from the shelf and transferred to the sampling plate by using the corresponding button on the control box. The shelf has 10 levels, with each level corresponding to one of the 10 numbered buttons on the control box. For instance, to analyze the #1 tray, place the plate at the collection position and press button C1.

**Fig. 15:**
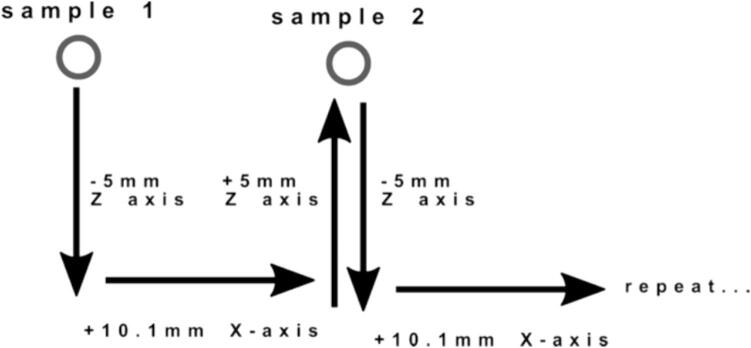
The plate movements for the analysis of the samples regarding the 1st sample aligned to the mass spectrometer inlet.

The collection position of the sampling plate is set at X0, Y54.1 and Z0 mm to receive the tray. During the analysis, the sampling plate is positioned relative to the first sample on the tray, and the analysis proceeds from left to right. In our experiments, the sampling plate was positioned at coordinates X8, Y14, and Z25 mm to align the first sample with the mass spectrometer inlet, with a separation of 5 mm between the paper tip and the MS inlet. To analyze subsequent samples on the tray, the position on the X-axis was increased by 10.1 mm, corresponding to the distance between each cartridge within the tray of the cartridges. The movement of the plate is depicted in Fig. 15.

## 7 Validation and Characterization

**Fig. 16:**
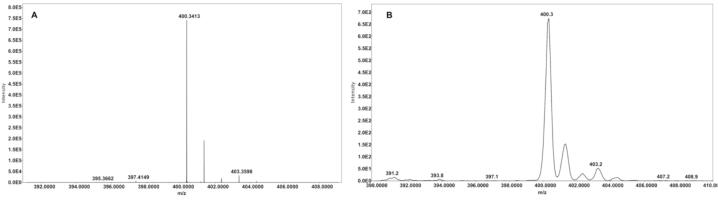
A) Mass spectra of palmitoylcarnitine measured on a Q Exactive Orbitrap and an LCQ-Fleet B) mass spectrometer using the Open SprayBot platform.

The platform's lateral resolution is 50 µm [18]. The platform was assessed using a Q Exactive Orbitrap and an LCQ Fleet (Thermo-Fisher Scientific, USA) (Fig. 16).

**Fig. 17:**
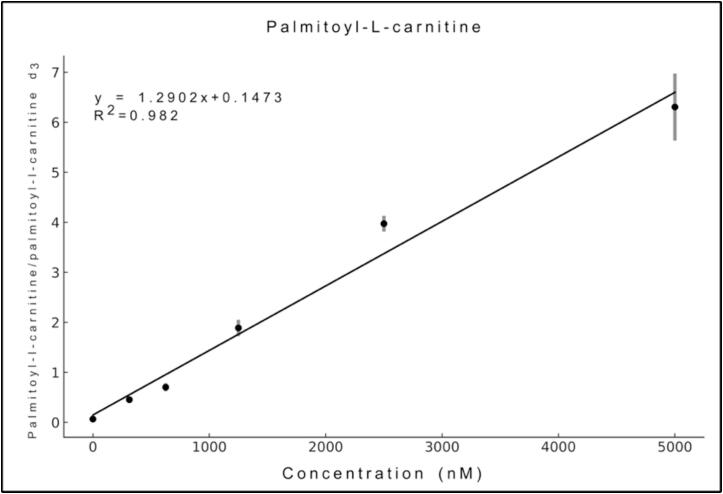
Calibration curve of palmitoylcarnitine by PS-MS.

To evaluate the system’s performance, a calibration curve of palmitoyl-l-carnitine was evaluated, encompassing concentrations from 313 nM to 5 000 nM, to reflect both normal and pathological levels. Palmitoyl-l-carnitine is a biomarker commonly tested in blood and urine for IEM screening. The calibration curve (Fig. 17) was measured in the QE orbitrap at selected ion monitoring (SIM) mode, in a mass range of 390 to 410 *m*/*z*. The system demonstrated good linearity for palmitoyl-l-carnitine (399.6 g/mol) when using a stable isotope labelled standard, palmitoylcarnitine ^2^H_3_ (402.6 g/mol) (Fig. 17) with a n = 5. Chemical reagents were purchased from Sigma-Aldrich (USA). Chromatographic paper Whatman #1 (Sigma-Aldrich, USA) was employed for testing the system. For all the experiments the solvent was applied using a syringe pump with a flow of 20 µL/min. Data were recorded in the LCQ Fleet with a capillary temperature of 200 °C, capillary voltage of 11 V and 120 V on the tube lens. The spray voltage was set to 3.5 kV.

Repeatability between analysts was also evaluated (Table 3) using palmitoyl-l-carnitine (1600 nM) and palmitoylcarnitine ^2^H_3_ (400 nM), as the stable internal standard, in a methanolic solution (n = 6) in an LCQ Fleet instrument. 5 µL of the solution was applied at 7 mm from the paper tip. Acetonitrile/water/formic acid (90/10/1) was used as the ionization solvent. All the solvents were HPLC grade acquired from Fermont (Mexico).

**Table 2:** Summary of bill of materials organized by platform components. ^a^ Canadian dollar.

Component	Number	Cost per unit (CAD^a^)	Total cost (CAD^a^)	Part number
**Support structure and shelf**				
3030 Extruded aluminum frame 650 mm (A)	4	58	232	−
3030 Extruded aluminum frame 340 mm (B)	6	19	114	−
3030 Extruded aluminum frame 340 mm (C)	7	19	133	−
3030 Extruded aluminum frame 300 mm (D)	1	16.5	16.5	−
3030 Extruded aluminum frame 400 mm (E)	1	16.5	16.5	−
T Slot L-shape 3030 6 mm	26	1.47	38.22	−
Aluminum (I) flat bar (1/4″ x 1.5″ x 12.5″)	4	12	48	−
Aluminum (II) L-shaped (1/16″ x 1″ x 1″ x 3.9″)	16	2.6	41.6	−
Aluminum (III) L-shaped (1/16 x 1″ x 1″ x 5.4″)	2	3.51	7.02	−
Aluminum (IV) L-shaped (1/8″ x 3/4″ x 3/4″ x 3.9″)	2	1	2	−
Aluminum composite black sheet (400 x 650 x 3 mm)	1	43	43	−

**Collector**				
2020 T slot extruded aluminum 400 mm	1	7.5	7.5	−
Pulley wheel for 6 mm with ball-end hex wrench	8	3.1	24.8	−
POM wheel plastic pulley	4	1.5	6	−
GT2 Pulley wheel	2	1.56	3.12	−
Belt GT2 2 mm x 6 mm wide	4	3	12	−
Mini linear rail guide MGN12H	3	28	84	−
stepper motor 1.8°	3	11	33	MT-1704HS168AF
Aluminum (V) L-shaped (1/8″ x 2″ x 1.5″ x 1.5″)	1	1	1	−
Aluminum (VI) L-shaped (1/8″ x 1″ x 1.5″ x 1.5″)	2	0.5	0.5	−
Aluminum (VII) L-shaped (1/4″ x 2.1″ x 1.5″ x 3.1″)	2	1.8	1.8	−
Aluminum (VIII) L-shaped (1/8″ x 2″ x 1.5″ x 1.5″)	1	1	1	−
Aluminum (IX) L-shaped (1/8″ x 1.5″ x 1″ x 1″)	1	0.75	0.75	−
Aluminum (X) L-shaped (1/8 x 1.5″ x 1.5″ x 1.5″)	1	0.75	0.75	−
Aluminum (XI) bar (1/4 x 2.5″ x 2.5″)	1	1.25	1.25	−
Aluminum (XII) L-shaped (1/8″ x 1.4″ x 1.7″ x 0.75″)	2	0.75	0.75	−
Aluminum (XIII) L-shaped (1/8″ x 2.5″ x 1″ x 6″)	1	1.5	1.5	−
Mounting L bracket Nema 17 42 for 2020 profiles	1	2	2	−
Mounting plate bracket Nema 17 42 for 3030 profiles	1	2	2	−

**Sampling plate**				
3030 Extruded aluminum frame 190 mm (F)	2	19	38	−
3030 Extruded aluminum frame 240 mm (G)	1	19	19	−
Mini linear rail guide MGN12H (185 mm)	4	28	112	−
Linear rail sliding block HGH15CA	4	17.5	70	−
Shaft support SK12	4	4.5	18	−
Linear motion rod 12 mm (350 mm)	2	13	26	−
Block bearing SCS1UU	4	4.5	18	−
Momentary micro limit switch SS-5GL2	3	0.89	2.67	−
Stepper motor 1.8° Nema 17 with leadscrew	4	50	200	MT-1703E168G2-350
T8 leadscrew nut	4	3.8	15.2	−
Aluminum (XIV) flat bar (1/4″ x 2″ x 10″)	4	0.16	0.64	−
Aluminum sheet metal (8″ x 8″ x 1/16″)	1	11	11	−

**Controlling system**				
SWITCH ROCKER DPST 20A 125 V	1	10.99	10.99	EG1531-ND
RepRapDiscount smart LCD controller + RAMPS Adapter	1	36	36	−
Button stainless steel (flat head) 12 mm	11	2.6	28.6	−
Arduino Mega 2560	1	52.5	52.5	−
7 segments display	1	1.3	1.3	−
Stepper motor driver controller DM542	2	22	44	−
RUMBA 32 board	1	164	164	−
Switching power supply (AC 110 V/220 V to DC 12 V 30A 360 W)	1	37	37	−
TMC2130 stepper driver	5	16	80	−

**Ionization system**				
Power supply (SRS, USA)	1	2 085	2 085	PS350 SRS
Coaxial cable SHV to SHV	1	165	165	−
Hex nut 0.250″ (1.4″) stainless steel	1			−
Syringe pump Chemyx F200	1	2 621	2 621	−
5 mL glass gastight syringe, PTFE Luer Lock (Hamilton) and 5 cm needle	1	70	70	−
PEEK capillary tubing 50 cm	1	69	69	−
DB15 male connector to solder 15 pins + box	1	7.46	7.46	−

**Table 3:** Precision evaluated for palmitoylcarnitine with the Open SprayBot in an LCQ Fleet.

**Precision**	**RSD %**
Repeatability	
Within-run precision (Analyst 1)	15.94
Within-run precision (Analyst 2)	18.84
Intermediate precision	
Within analysts	17.39

To test the HT of the platform, a methanolic solution of palmitoylcarnitine (800 nM) with its stable internal standard (400 nM) was measured 120 times using individual and new paper strips of Whatman no. 1. Acetonitrile/water/formic acid (90/10/1) was used for this experiment and the LCQ Fleet instrument was employed. The relative standard deviation (RSD) was 17.87 %.

## 8 Conclusion

This work introduces the Open SprayBot platform, a low-cost, open-source, and nearly fully automated PS-MS system. In principle, the Open SprayBot can be attached to any mass analyzer with an electrospray ionization interface. Before any mass spectrometry hardware and software modifications, users should communicate with their provider to avoid the risk of voiding the system's warranty.

The particular setup for enabling the external paper-spray source depends heavily on the mass spectrometer model. Possible solutions are software settings (for example, choosing a compatible ambient ion source), a hardware dongle for simulating the presence of an ion source, or adopting an ion source (e. g., perforating the ion source chamber and passing an ion transfer tube).

The Open SprayBot is a design to facilitate high-throughput (HT) PS-MS analysis. This platform offers a cheaper, easily modifiable, highly repairable, more accessible, and far more user-friendly platform for the PS-MS community. The system’s robustness, accuracy, and reproducibility, measuring clinical conditions of a biomarker of disease on two different MS platforms in two different countries (Canada and Mexico) was demonstrated. The system has significantly reduced the need for manual intervention in PS-MS analysis. The next step in the ongoing development is to achieve full automation (specifically in terms of paper cutting and paper/cartridge placement).

The cost of this platform, including both the sampling module and ionization module, is approximately 20 times less than commercial instruments. An external power supply accounts for 30 % of the total cost of the system, but this could be eliminated by taking the power from the mass spectrometer. Another 30 % of the total cost corresponds to a syringe pump, however, this is a common instrument in mass spectrometry laboratories.

Therefore, the Open SprayBot presents a promising alternative to existing HT PS-MS platforms. Our goal in creating this system and disclosing its construction details is to advance HT mass spectrometry analysis for low-cost disease screening and/or drug screening from blood or urine spots.

## CRediT authorship contribution statement

**Nancy Shyrley García-Rojas:** Writing – original draft, Methodology, Conceptualization. **Héctor Guillén-Alonso:** Visualization, Methodology. **Scott MacKay:** Writing – review & editing. **Claudia Torres-Calzada:** Writing – review & editing. **Leonardo Daniel Soto-Rodriguez:** Methodology, Conceptualization. **Robert Winkler:** Writing – review & editing, Supervision, Resources, Funding acquisition, Conceptualization. **David S. Wishart:** Writing – review & editing, Supervision, Resources, Funding acquisition, Conceptualization.

## Declaration of competing interest

The authors declare that they have no known competing financial interests or personal relationships that could have appeared to influence the work reported in this paper. RW is CEO and shareholder of Kuturabi SA de CV.
